# Toward mass customization of animal trackers by design automation

**DOI:** 10.1371/journal.pone.0342071

**Published:** 2026-02-04

**Authors:** Patrick Beutler, Troels Gregersen, Joël Roth, Nina Richter, Mirko Meboldt, Martin Wikelski, Timm A. Wild

**Affiliations:** 1 Inspire AG, Zurich, Switzerland,; 2 Product Development Group Zurich pd|z, ETH Zurich, Zurich, Switzerland; 3 Department of Migration, Max Planck Institute of Animal Behavior, Radolfzell, Germany; 4 Department of Biology, University of Konstanz, Konstanz, Germany; Sreenidhi Institute of Science and Technology, INDIA

## Abstract

Animal-borne tracking devices (bio-loggers) are established instruments for researching animal behaviour. However, commercial animal trackers are rather standardized and not perfectly adapted to species-specific requirements. Although species-specific solutions are developed, customization effort is high and requires detailed engineering know-how. Furthermore, the development process brings multiple challenges across the process chain and uncertainties for untested species may require iterative refinements in the early design phase. This interdisciplinary study provides a vision of how to enable mass customization of animal trackers through a web-based design platform. The platform involves biologists in engineering processes, makes custom designs accessible to the community, and enhances reusability. Knowledge-based engineering and design automation algorithms are central platform elements, and they automate engineering processes from requirements to the electronic component selection and generation of 3D-printable housing geometries. The animal tracker housings are manufactured using low-cost 3D-printing (additive manufacturing), which offers high flexibility in terms of producible geometry and batch size. Furthermore, this study presents a design automation prototype that implements core functions of the vision to demonstrate the feasibility of automatically generated animal trackers. The software architecture of the design automation prototype and the intermediate algorithm steps are described as open source. To demonstrate the functionality of the design automation prototype, the animal tracker housings of three species are successfully generated and produced. The algorithms take less than 50 seconds to generate the three housings. This demonstrates, how the automation eliminates bottlenecks in the development process and thus greatly reduces efforts for customized animal trackers. The full realisation of the vision can eventually empower biologists to design animal trackers without the involvement of engineers.

## Introduction

Animal trackers play a crucial role in modern wildlife conservation, providing essential data to understand animal behaviour, migration patterns, and habitat use. They allow the monitoring of species in real time, offering insights that help protect endangered populations and manage ecosystems more effectively [1–3]. As the impacts of climate change and habitat loss intensify, animal trackers become increasingly important tools for preserving biodiversity [4–7]. Researchers are striving for a global network of connected animals under the term *Internet of Animals*, which could enable the observation of even more complex natural processes [8]. At the beginning of the wildlife tracker revolution in the 1960s, only a few species could be tagged due to the large size and weight of the first radio transmitters. Since then, technological advances in consumer electronics, miniaturization, and manufacturing technology have pushed the technical boundaries of animal tracking devices. The current state-of-the-art animal trackers only weigh a few grams [9,10]. Consequently, the limiting technological factor is no longer the size and weight of animal trackers, but rather the limited resources available for developing new devices for specific species.

**Fig 1:**
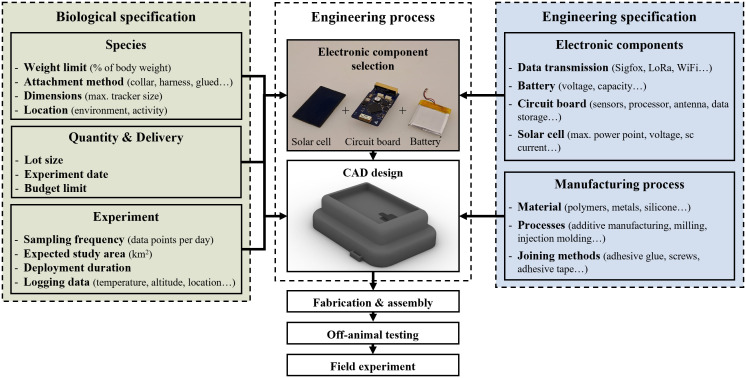
Interdisciplinary specifications for species-specific animal trackers. Biological specifications include requirements regarding species, quantities and experiments. Engineering specifications include selection of electronic components and manufacturability.

The hardware development of species-specific animal trackers represents an unprecedented case for mass customization. Species-specific requirements are driven by the immense diversity in species physiology, behaviour, environmental conditions, and specific attachment to the animal’s body. Close collaboration between biologists and engineers is required (Fig 1). Biologists specify the requirements for each species, such as body size, weight, movement patterns, and environmental conditions. Meanwhile, electronic components that meet all requirements and constraints must be selected. This selection requires balancing of factors such as sensor selection, battery life, and data transmission capabilities. Based on the electronic components, a housing must be designed in a computer-aided design (CAD) program that accommodates the components, holds them in place, and protects them against external influences. Animal trackers must withstand challenging environmental conditions, such as varying atmospheric pressures, intense solar irradiation, fluctuating humidity, temperature levels, and continuous mechanical stress caused by animal movements. Furthermore, these devices must not harm or injure animals. This can be achieved with lightweight devices, and well-evaluated noninvasive attachment to minimize discomfort and allow animal engagement in natural behaviour [4,11,12]. Reducing both behavioural disturbance and physical impact is essential for the ethical deployment of bio-loggers [13,14].

**Fig 2:**
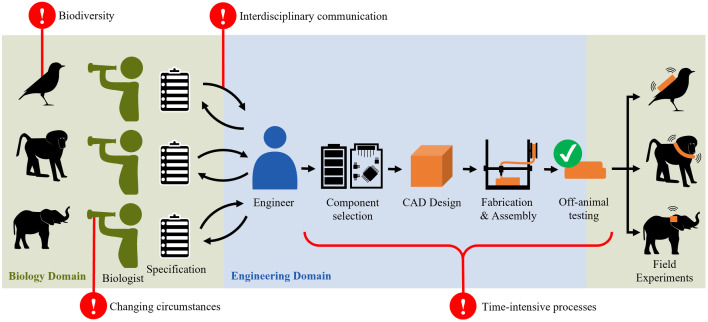
Challenges in the development process of animal trackers. Key challenges in the process from observation to field experiments are the large biodiversity, changing circumstances in nature, interdisciplinary communication, and time-intensive development and manufacturing processes.

The hardware development of products where the design, engineering, and manufacturing processes are tailored to specific customer requirements are referred to engineer-to-order (ETO) products [15–18]. The variation in requirements in ETO products presents substantial challenges throughout the development process of hardware components that are also relevant to animal trackers (Fig 2): Challenge one is the requirement for highly flexible engineering solutions to cover large design varieties [19–21]. In animal trackers, the vast biodiversity between species demands a wide variety of tailored products. For example, biologists often specify that animal trackers must not weigh more than 5% of the body weight of an animal, and the attachment methods can largely differ depending on the species [1,22]. Challenge two are requirement changes in ETO products that often lead to multiple design iterations, driving up costs and causing delays [23–26]. For animal trackers, the situation differs: requirement changes in later development phases are usually not feasible due to small number of devices produced. However, uncertainties during the early design phase are common when working with species that have not been studied before [27]. Initial deployments may reveal unexpected reactions or attachment issues, requiring early-phase refinements and emphasizing the importance of formalizing species-specific knowledgd. Challenge three is effective communication in interdisciplinary product development processes for ETO products [28,29]. For animal trackers, extensive communication between biologists and engineers is often needed to clarify all requirements and technical capabilities. This creates a dependency of the biologist on the engineer, which leaves the biologist without a working solution when the collaboration is discontinued during an ongoing project. Challenge four for ETO products is to be competitive with mass-produced alternatives in terms of delivery time and costs, which are usually significantly higher [23,30]. Likewise, the mass-customization of animal trackers can lead to extended production timelines and increased expenses. The lack of engineering knowledge tracking limits reuse of design decisions from previous projects.

Since these challenges are difficult to overcome, commercial providers focus on a few products that can be deployed to multiple species. However, the lack of adaptation could lead to discomfort and even injury to animals, and their applicability may be limited. Researchers and hobbyists who have no access to customized solutions also deploy do-it-yourself solutions that involve condoms or nail varnish for waterproof animal trackers [31–33]. Such approaches can lead to discomfort for the animals, do not guarantee repeatable quality, and are not scalable as they involve high manual efforts. Therefore, novel solutions are needed that make species-specific animal tracking scalable, cheaper, reduce manual efforts, and increase the reusability of both biologist and engineering knowledge [34].

This paper makes two contributions to enable mass-customized animal trackers. The first contribution is a vision for a web-based product design platform that can automatically generate production-ready animal trackers based on species-specific requirements. In this vision, the previously mentioned challenges in the ETO process chain of species-specific animal trackers are addressed. The vision is based on two key process steps that should be considered for an efficient ETO product platform: the engineering and the manufacturing process [15,35,36].

Animal trackers are highly suitable for production using additive manufacturing (AM), also known as 3D printing [9,10,37–40]. The advantages of AM, such as short lead times, no tooling costs, batch size independence, and design freedom, can be fully exploited [41–43]. For the engineering processes, the vision includes a high degree of automation by applying well-known engineering approaches: design automation (DA) and knowledge-based engineering (KBE). DA refers to the use of software and computational methods to automate the engineering design process, from initial concept to detailed design and optimization [44–48]. DA reduces manual efforts, accelerates product development processes, and enhances design consistency by automating design tasks. KBE leverages structured knowledge and rules to automate design decisions [44,47,49–51]. KBE systems use formalized knowledge encoded into software tools to guide decisions in the engineering process. The efficiency and quality of engineering processes are improved by ensuring that the results comply with best practices and standards. The combination of highly flexible AM processes and the automation of engineering processes by KBE and DA are enablers for the efficient supply of ETO products [30,51,52]. The KBE and DA algorithms run in the background (back-end) and users can interact with them through user interfaces (front-end) provided on a web-based design platform. Our vision describes how these technologies can be applied to species-specific animal trackers and what elements are required.

The second contribution of this paper is an automated housing designer prototype that automatically designs species-specific animal trackers based on the manual selection of electronic components. The DA prototype is developed as a minimal example to demonstrate the implementation of key functionalities of the vision. The DA prototype is based on previous work, which considers both the functionality of the component and the manufacturing constraints of AM in automated design processes [53–55]. A strategy for the reuse of purchased parts in different assemblies is also adopted from an engineering application by implementing a part library for electronic components [56]. The DA prototype is applied to three different use cases to demonstrate the diversity of the resulting designs for three different species (Section 4). The generated animal tracker housings are 3D printed and assembled with electronic components. Finally, the next steps toward a full realization of the vision are described, and the bio-logging community is encouraged to actively participate in this process.

### Vision

**Fig 3:**
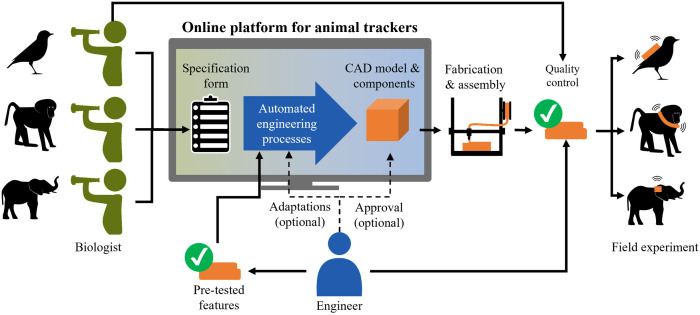
Vision for automated development process of animal trackers. An online platform for the auto-generation of animal trackers by biologists is the core component for the automation of engineering processes.

The central element of our vision for improving the development process of species-specific animal trackers is a web-based design platform (Fig 3) that is accessible for research groups around the world. The platform is a website that provides graphical user interfaces for both biology and engineering specifications. The platform has access to algorithms that can automatically generate the 3D CAD of animal tracker housings. The biology interface allows to specify the biological experiment with species-specific information and experimental requirements. This allows for a standardized specification form of the experiments that is complete enough to automatically generate an animal tracker. Based on this specification, KBE algorithms select electronic components (battery, circuit board, and solar cell) that can be used for the specified experiment. In this process, the KBE algorithms access the data from existing animal trackers of similar species to decide on electronic components. Additionally, the reusability and qualification of electronic components in a component library facilitates a wide variety of animal trackers.

Furthermore, the 3D CAD geometries of the housing are automatically generated by DA algorithms. The auto-generated geometries of the housing, the selected components, and details such as a cost and device mass estimates are provided directly to the biologist. This allows biologists to design the first version of an animal tracker independently of an engineer and receive instant feedback on the technical feasibility of the project. The engineering interface allows adjustments to the design in terms of detail dimensions and an approval of the final housing design for production. These changes can be made by biologists via the engineering interface, as no specialist knowledge is required, or optionally shared with an engineer for adaptation or approval. This reduces biologists’ dependency on engineers, and engineers spend less time on repetitive tasks and can focus on maintaining the platform, extending individual features, and testing the produced animal trackers. This means a formalization of engineering know-how with pre-tested features that can extend the DA workflows. Pre-tested design features can significantly increase the quality of the final trackers as validated features are reused [57]. The production of housing components is done using AM. Thanks to the improvements in AM processes and their suitability for decentralized production, it is possible to produce the parts through a global network of service providers, which increases the flexibility in the production step [58,59]. It is important to note that the responsibility of the generated animal tracker and the field experiments is still with the biologist. This includes quality control and testing of ingress protection (IP) and attachment breaking strength prior to deployment. Furthermore, the biologist has to ensure that ethical criteria are fulfilled and the online platform must be seen as a supporting tool.

The combination of a simplified user-friendly web interface with KBE algorithms for the selection of electronical components and DA algorithms for the automated design of the housing solves multiple challenges in the development process: (I) the large variety of animal trackers can be solved by modularity and the component library for electronical components, (II) design changes due to changing circumstances may still occur but can be implemented quickly, (III) biologists become more independent of engineers and interdisciplinary communication can be standardized, and (IV) ETO products can be offered with competitive delivery times and costs through automated engineering processes and additive manufacturing. Involving biologists into the design process further allows them to give feedback on the design, request new design features for the platform, and identify design mistakes that might not be evident to engineers.As biologists gain more experience, they can eventually be empowered to design animal trackers without the involvement of engineers. To have a real impact, such a platform should be able to create most bio-logging devices.

**Fig 4:**
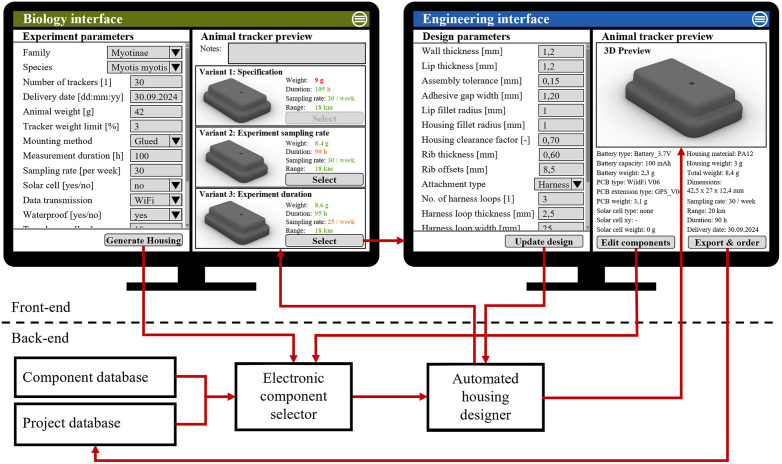
Software architecture of the online platform. The front-end of the online platform includes a biology and an engineering interface to modify specifications. The back-end includes databases for electronic components and past projects, and two software blocks for the selection of electronic components and the generation of housing geometries.

Fig 4 shows a detailed draft of the web-based design platform with a front-end and a back-end visualization. Two interfaces are envisioned for the front-end, which are aimed at biology and engineering specifications, respectively. The biology interface allows users to specify the experiment by providing information on the species by biological classification (class, order, family, genus, species), experiment duration, required sensors, measurements per time, maximum device mass, and attachment method. The biological specification must be complete to derive quantitative evaluation criteria for the final design, such as a maximum mass. After completing the specification, a suitable electronics and power source combination is chosen, and the housing is automatically generated. If the specification of the experiment is technically not feasible, the tool can generate design variants and display the limitations. As an example, a specification may describe a 72-hour experiment with 30 GPS points per day, and the required battery would make the tracker too heavy for the animal. Alternatives are automatically suggested with shorter experiments or fewer data points. The biologist can select the preferred version and proceed to the engineering interface to adjust the design or share the project with a colleague.

The engineering interface focuses on the selection of the electronic components and the detailed dimensions of the housing geometry. When editing the housing, the automatically selected components can be adjusted, and individual design parameters can be changed. For example, the wall thicknesses can be adapted to the manufacturing process and material, or tolerances for assembly can be changed. In this way, the engineering interface is used to ensure that the components can be manufactured and assembled. Afterwards, the final geometries can be exported, revised and released for manufacturing.

The back-end, the part of the program that contains the logic and is invisible to the user, contains two databases and two software packages. The *component database* is set up for electronic components such as batteries, circuit boards, and solar cells. Elements of this database must include the detailed geometries of the electronic components and their technical specifications, such as the nominal voltage, the capacity of a battery, or the dimensions of a solar cell. This database must be publicly accessible so that all users can find the most suitable components for their experiments. Therefore, the component database is set up as an online marketplace where manufacturers of batteries, circuit boards, or solar cells can place their products. The order of these products can be initiated through integrated web shop tools, so that manufacturers also benefit that their components are available in the library. The *project database* stores information from current and past projects. Past projects contain information about the used components, the specification of the experiment, and the actual measurements. Users can decide themselves whether a project should be shared publicly or kept private. Animal trackers that are involved in published scientific work should be made accessible for transparency and to promote related publications.

Software packages in the back-end can be subdivided by their function. The first package determines the most suitable electronic components for a specification: the *electronic component selector*. As consumer electronics have become cheaper and mass-produced, the electronic component selector chooses from verified and tested off-the-shelf components that are added to the component database. There are two approaches to selecting the electronic components: an analytical approach and a data-based approach. The analytical approach involves a formula that uses the information in the specification, such as the number of measurement points and the duration of the experiment, as variables. When the data transmission technology is determined and solar cells are applicable, a solar cell and a battery are determined. With the data-based approach, a neural network is trained to automatically determine suitable components based on data from past projects. The data-based approach helps when an experiment can neither be fully defined nor past projects can be used as references. However, a significant number of documented projects are needed to train such a model robustly.A key function of the electronic component selector is to estimate power consumption and battery life for a defined experimental setup, as these factors directly influence tracker weight and size.

The second program package, the *automated housing designer*, automatically generates the 3D CAD models of the housing. The geometries must be designed based on the selected components and the biologist’s specifications. The automated housing designer creates CAD models based on predefined CAD functions considering assembly, material choice, AM design constraints, gluing processes, and tolerances. This not only increases consistency of quality, but also enables standardized testing of the predefined CAD functions across products. For example, a housing usually consists of two parts that need to be joined after assembly with electronic components. This joining method and the related CAD functions can be thoroughly tested for watertightness and strength to create a prevalidated feature that is safe for reuse in future projects [57]. This allows the separation of development and testing of new validated CAD features and the development of species-specific animal trackers, which further reduces time and cost. The automated housing designer saves a significant amount of time in the development process and can be crucial to the success of an experiment when dealing with critical deadlines and design changes. Time savings in component selection and design engineering processes also enable the creation of more product variations, and can therefore be seen as a key enabler for scaling up animal trackers for more species.

### Prototype

**Fig 5:**
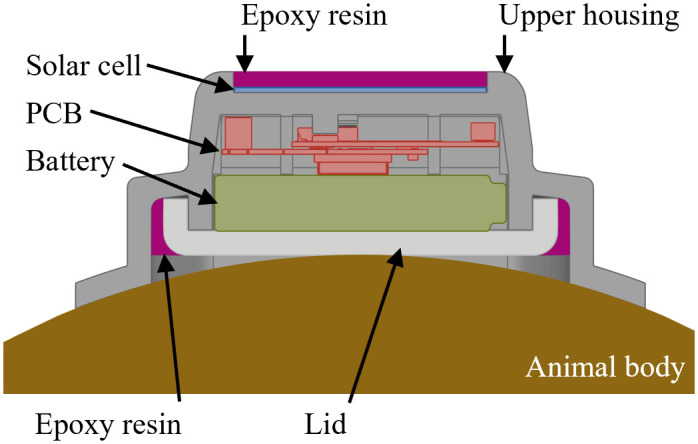
Part architecture of the animal trackers. All trackers considered in the design automation prototype share the same potential types and spatial order of electronic components: battery, PCB, and solar cell. The battery is always closest to the animal’s body.

The automated housing designer is a key element of the vision to enable automated generation of CAD models of animal tracker housings. The purpose of the prototype is the automated generation of animal tracker housings with a predefined product architecture (Fig 5). This product architecture specifies which electronic components can be used and how they are arranged spatially: a battery is placed close to the animal’s body, and a circuit board with a microcontroller, sensors, data transmission modules, and antennas is placed on top of the battery. Solar cells are optional for day-active animals and are placed on top of the electronics. The housing consists of two parts: the upper housing, which holds all the components, fixes them in place, and contains the attachment geometries, and a lid, which is required for closing the housing watertight after assembly. The current method for joining the upper housing and the lid utilizes a groove filled with liquid epoxy resin. If a solar cell is used, it is bonded and sealed with transparent epoxy resin.

**Fig 6:**
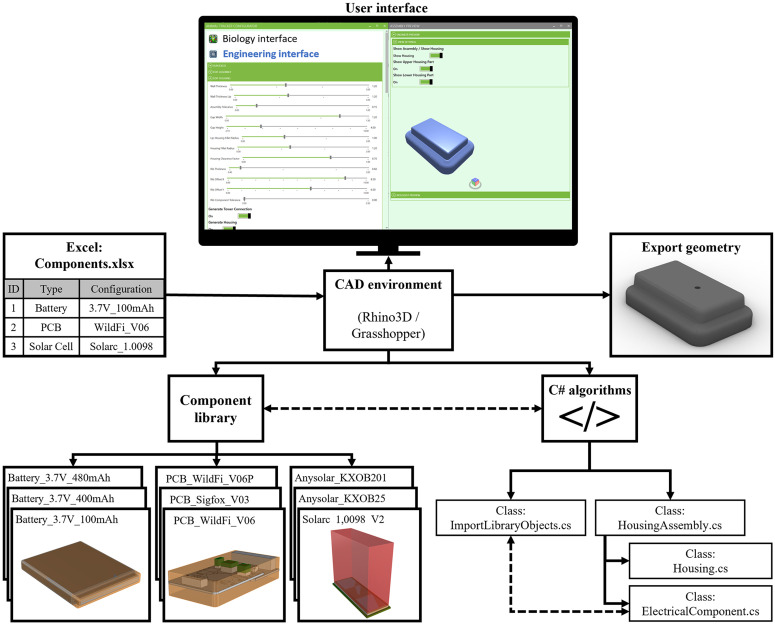
Software architecture of the DA prototype. The electronic components are specified in an excel file. The excel file is imported into a CAD environment with a graphical user interface. The user interface allows manipulation of design parameters. The CAD environment has access to electronic components in a component library and C# algorithms that generate the geometries.

The software architecture of the developed DA prototype is shown in Fig 6. The battery, circuit board, and (optional) solar cell can be specified manually in an Excel sheet. Electronical components stored in the component library are available for this selection. In this prototype, the component library is simplified as a local folder with a specified path. The components stored in the component library are saved in a Rhino3d file and contain the detailed CAD models of the electronical component, their bounding box, geometries of interfaces that are to be retained and geometries of openings that are required for the respective component. To ensure that the corresponding geometries can be found, they must be stored on specific layers, and the files in the library must follow a naming convention. The tasks of the automated design process are performed by C# algorithms. The most important API used in the C# algorithms is the *RhinoCommon API*, which allows access to the CAD functions of *Rhino3d* (McNeel & Associates (TLM, Inc), USA) within the code. The C# algorithms are developed using an object-oriented programming (OOP) approach and are thus organized in multiple classes. A class for *Components* contains functions and properties that are used for all electronic parts. These are, for example, detailed geometries, bounding boxes, or type of electronic components. Another *Housing* class contains properties and functions for creating the actual housing. For example, design parameters such as wall thicknesses or fillet radii are defined in this class, and functions for creating the housing geometry or different attachment options on the animals are defined. Rhino3d is a CAD software with a graphical programming interface called Grasshopper. Rhino3D and Grasshopper are central elements of the prototype. First, the C# algorithms are executed from a Grasshopper file, and second, the C# algorithms access the CAD functions of Rhino3d in the code via the RhinoCommon API. For the prototype, a user interface was created using the Human UI Grasshopper plugin, which allows the modification of design parameters via interactive elements such as number sliders. The current prototype can generate 3D geometries in the STL and STEP file format, which can be directly used for production using additive manufacturing processes.

**Fig 7:**
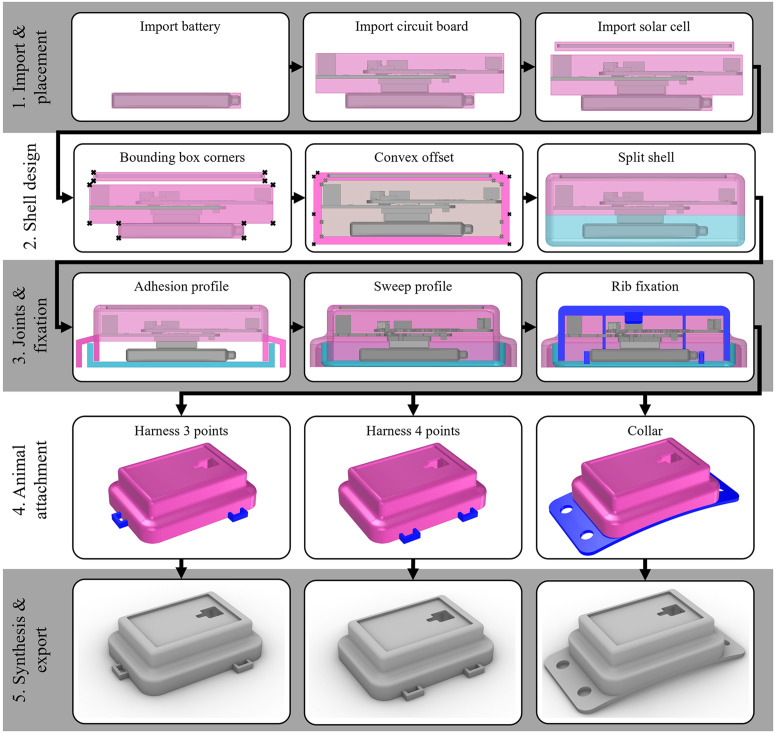
Automated design steps in the software prototype. 1. Import and placement of electronic components, 2. Shell design around the components, 3. Design of adhesive joint and ribs for fixation of electronic components in the housing, 4. Selection and design of an animal attachment, and 5. Design synthesis and export of 3D printable housing geometries.

The automated design process is not a black box that generates a fixed result. It is a tool that can generate a geometry with standard parameters, but can also be modified if necessary.Hence, users have the option of customizing the design to their preferences using number sliders in the user interface that control design parameters such as the wall thickness of the housing. The DA process executed by the C# algorithms consists of five steps with several substeps, which are described in more detail hereafter. Fig 7 shows the geometries that are generated in the intermediate steps of the algorithm for an improved illustration of how the code works.

In the first step, the Excel file with the component selection is read and the required instances of the C# classes for the housing are initialized. The component geometries are imported from the component library and assigned to the initialized class objects. The electronic components are positioned in a specific order: the battery is placed first, then the circuit board, and finally the solar cell, if there is one. When positioning, components are placed centrally on top of each other with reference to the bounding boxes of the electronic components. If the solar cell is mounted from the outside, the algorithm calculates the distance needed to maintain a minimum wall thickness.

In the second step, a convex pyramidal shell is calculated. The corner points of all bounding boxes are taken and a calculation defines the corner points needed for a convex shape. Furthermore, it is ensured that the envelope has a pyramidal shape, which means that the cross-section must decrease towards the top. This is necessary, since the components are mounted from the bottom side during assembly. All edges where an animal could get caught or where dirt can deposit should be avoided by this shape. The shell is given an offset of the wall thickness and split into a top and a bottom part.

In the third step, an adhesion profile is generated, of which all dimensions can be changed in the UI. This profile defines the adhesive gap with which the two housing halves can be glued together watertight after assembly. The profiles of the upper and lower part of the housing are swept along the contour of the created shells. To prevent components from sliding within the housing, additional ribs are created to hold them in place.

In the fourth step, the geometry needed to attach the tracker to the animal is generated. The prototype provides four different options that can be selected. Option zero is used when the tracker can be glued directly onto the body of the animal and does not require any further geometric features. For birds, harnesses and leg loops are often used to attach an animal tracker to the back of the bird by fixation to their wings or legs. For this attachment approach, a version with three (option 1) and one with four mounting points (option 2) is implemented in the prototype. In mammals, animal trackers can be mounted around the neck in the form of a collar. For this attachment type, there is an implementation in the prototype with a curved bottom surface and two holes per side as interfaces to the collar (option 3). The user can modify all dimensions of the attachment interfaces to customize them for a specific species.In the fifth and last step, all Boolean operations (unions, subtractions, etc.) are performed to obtain the final geometries, which can be exported as an STL or STEP format.

Afterwards, the project is saved as a separate file, including the selection of electronic components and the values of all design variables. This project file can be reopened and adapted if changes need to be implemented.

### Use cases

**Fig 8:**
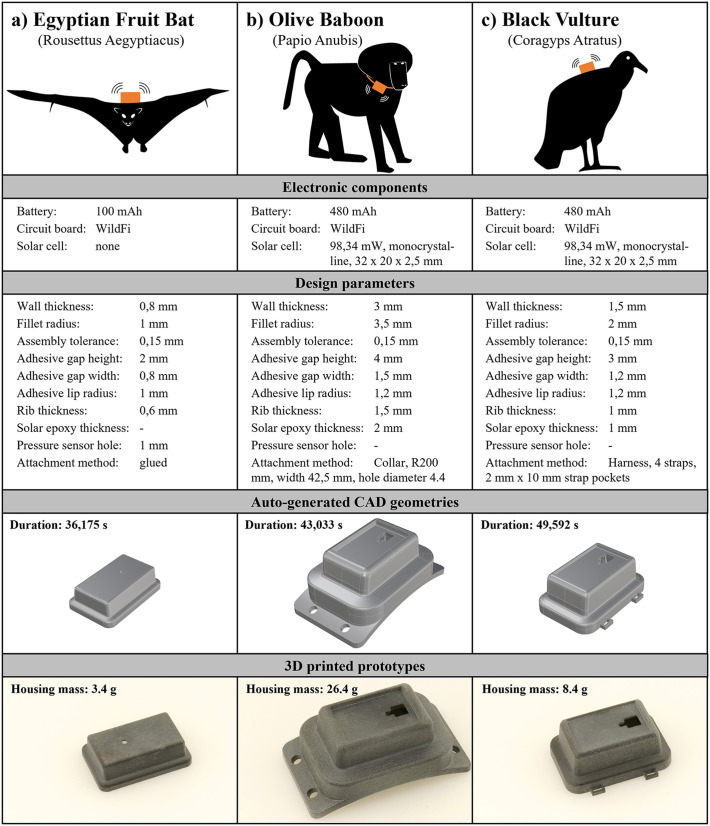
Overview of three use cases: (A) Egyptian fruit bat, (B) Olive baboon, and (C) Black vulture. The following information is provided for each use case: selected electronic components, design parameters, generated CAD geometries, and 3D printed housings made of PA12.

To demonstrate the feasibility of the DA prototype, it was applied to three use cases that are inspired by real experiments. The selection of electronic components is performed manually and is currently limited, as the number of validated components is small. For example, only one circuit board with the WildFi board is implemented [60]. Nevertheless, the variability of possible designs can be shown in the three cases based on the different animal requirements and the selected design parameters. The first experiment refers to the observation of Egyptian fruit bats (*Rousettus aegyptiacus*) in Cyprus (Fig 8A) [61]. As the animals are small, the trackers should be particularly light and small. The benchmark housing of the reference study has a mass of 7.7g. Due to their nocturnal activity, no solar cell is used, and because the trackers are glued to the back, no attachment geometries are required. Design parameters are set to minimal wall thickness and small adhesive interfaces. To measure pressure changes via a pressure sensor, a hole is automatically generated in this housing, onto which a pressure vent can be mounted. The second use case creates a housing for olive baboons (*Papio anubis*) in Kenya (Fig 8B) [62]. Due to the high strength of baboons and the rough interaction with other animals, this housing is built very solidly with a large wall thickness. The animal tracker is attached via a collar. The third use case is designed for black vultures (*Coragyps atratus*) in the Andes (Fig 8C) [63]. The housing for this large bird can be built more robustly than the one for the bats and contains a solar cell. It is attached to the back of black vultures with a harness [64]. In a reference study, Andean condors (*Vultur gryphus*) are tracked with 100g animal trackers [63].

As shown in Fig 8, the housings for all three use cases were automatically generated using the DA prototype of the automated housing designer. The auto-generation of the animal tracker housings takes between 35 and 50 seconds on a Windows notebook for all cases. Further information on the computer system and the performance of the DA prototype are provided in S1 Fig. The software prototype was developed within five months with a total development time of approximately 790 hours. The time required to manually design a 3D-printable animal tracker housing takes between two and five hours, depending on the complexity of the design for one development iteration. Usually, it takes at least three iterations until the final design is reached. Breakeven is achieved after the creation of 79 animal trackers if an average manual design time of ten hours is assumed for all iterations. Thanks to the object-oriented approach with the individual C# classes, new functions and improvements in the existing code can be implemented with little effort.

**Fig 9:**
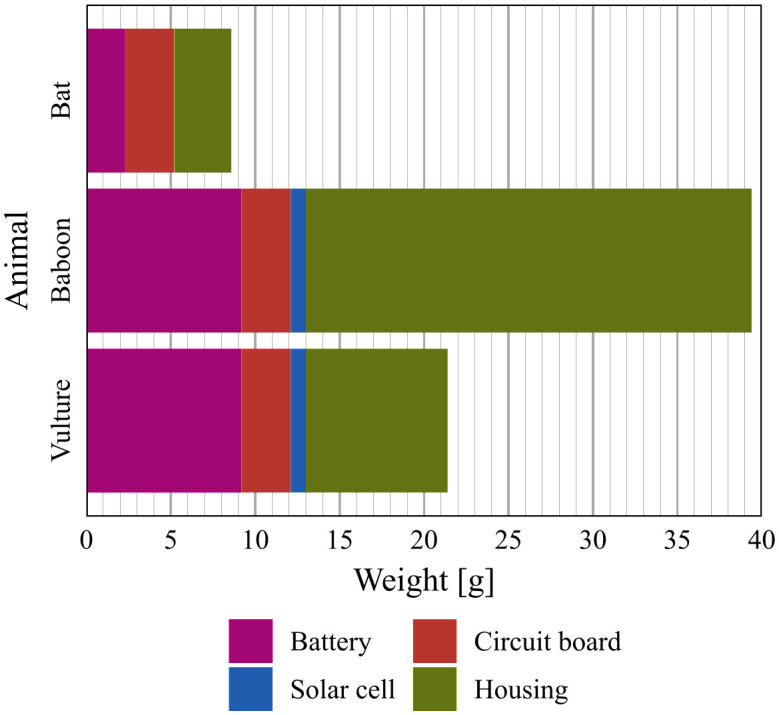
Mass of animal tracker prototypes. The total mass of the animal trackers consists of the housing and electronic components.

The models were used without manual adaptations to produce physical prototypes using AM (3d printing). Single-step binder jetting on polymer materials (BJT-SSt/P, also multi-jet fusion) was selected as AM process due to its high accuracy and good mechanical properties [65–68]. The parts were produced with PA12 material on an HP Jet Fusion 4200 printer. This process and material also enable low-cost production. With an order size of 30 pieces, the housing for the bat use case was offered with a price of 0.99 euros per piece by a 3D printing service provider (weerg.com, 29.11.2024). Fig 9 gives an overview over the weights of the individual components of the animal tracker prototypes produced. It is obvious that the design of the housing can have a significant influence on the total weight of the animal tracker and that lightweight housings can be achieved with optimal design parameters. The figure neglects the weight of the epoxy adhesive and the cables between the electronic components. The animal tracker prototypes of the use cases were not deployed to animals.

## Discussion

Smaller and cheaper electronics have enabled the use of animal trackers for an increasing number of species and this technology trend has intensified research activities and the use of technological solutions in recent years [5,8]. Since animal trackers should not disturb or harm animals, species-specific animal trackers should be used [4,34]. This is an unprecedented case of mass-customized products that must comply with species-specific requirements such as attachment method or maximum weight [11,13,14]. The hardware development process of such products has multiple problems: lack of resources, long lead times, expensive labour carrying out manual tasks, and development iterations are often unavoidable. This publication addresses this problem with two contributions. First, a vision is described that shows how the hardware development process for animal trackers can be automated considering requirements from both the biology and the engineering perspective. Second, the feasibility of the vision is demonstrated by a first implementation of the key element: the automated housing designer.

The proposed vision solves several challenges in the product development process for animal trackers such as high flexibility that can cover a large range of different species, facilitating fast changes through design automation, unified interdisciplinary communication, reuse of engineering knowledge through prevalidated features, and reduced development time. The envisioned platform meets the needs of both biology and engineering requirements by providing two specific user interfaces tailored for the respective domain. Biologists can receive direct feedback on the technological feasibility of their experiment through the automation of engineering processes. If the experimental design is infeasible with available bio-logger electronics, alternatives are suggested by the platform. This automation includes the automated selection of electronic components (including circuit boards, batteries, solar cells, antennas, etc.) and the automated generation of 3D-printable housings. Consequently, biologists are involved in the development process of species-specific bio-loggers and experimental design knowledge can be reused. The main benefit for companies and engineers is the reduction in development time, where their focus can shift from repetitive manual CAD tasks to developing and testing new features and evaluating new electronic components. Additionally, the standardization within the tool may help to achieve repeatable device quality and reduce the necessary effort for individual tests before deploying an animal tracker. The high degree of automation in engineering processes could ultimately allow biologists to generate animal tracker housings without the support of engineers. The approach of a publicly accessible platform allows the scaling up of the number of tracked animals per project as time can be reduced, and animal tracking devices can become cheaper. A web-based platform can help the entire community to intensify research and collaborations in the field of animal tracking between organizations and independently of geographical borders. In this way, the platform can boost the scientific activities of bio-loggers.

The design automation prototype presented demonstrates the feasibility of the key element of the vision: the automated creation of 3D CAD geometries of animal trackers. The engineering design process is fully automated, so that only the selection of components and final approval of the autogenerated design must be done manually. The 3D CAD geometries of the housing and the assembly with the electronic components are generated automatically. The output geometries can be manufactured using additive manufacturing, assembled and sealed with epoxy resin. The architecture of the prototype involves all components of the vision but with simplified elements. For instance, a component library with electronic components is implemented as a server folder and not yet as a globally accessible database. In addition to a detailed description of the prototype algorithms, the paper also presents the application of the prototype on three different use cases. Due to the variability of the species in the use cases, it can be shown that the prototype can generate animal trackers for diverse animals and multiple attachment methods. The computation of the use case housings took less than one minute. Thus, the time to customize bio-loggers can be greatly reduced compared to a manual design engineering process that can take multiple hours to days. The time savings increase even more if the design must be iterated. Using our automated housing designer for animal trackers eliminates the need for DIY attachments and sealings, such as condoms, and thus can increase animal well-being. The housings of the use cases were produced using a 3D printing process that enables low-cost production. Consequently, housings can be manufactured for less than one euro/pcs at an order size of 30. However, a reliable, decentralized production of 3D printed housings requires the certification of service providers that deliver parts with consistent quality.

As this publication presents a vision for a tool describes how animal trackers can be developed in the future on an interdisciplinary platform, there are many opportunities for further research. This work focuses on the development process of hardware components, but similar challenges arise in software development for animal tracking, circuit boards, data transmission and data analysis [5,60,61]. The design automation prototype is a minimal example, which demonstrates the feasibility of an automated process chain from the design to manufactured prototypes. Improvements and extensions to the current design automation prototype can be made for additional attachment methods, such as ear tags, increased robustness, reduced computational time, or alternative joining options to adhesive epoxy resin. Another additional feature is the option for more aerodynamic housings, which can reduce drag on flying or swimming animals, but also increase the tracker size. Nevertheless, the bio-logging community is encouraged to communicate its input on useful extensions via the discussions capability in the GitHub repository of the source code. The code should be further improved to achieve higher modularity, making it as easy as possible to add new features, such as additional attachment methods. Furthermore, new components (batteries, circuit board, and solar cells) should be integrated into a database (ideally by device engineers). Based on this, a first version of an automated component selector can be developed, which can determine the most suitable electronic components based on the definition of an experiment. The logic for this must be developed and tested with use cases. Hence, this work includes a first draft for a platform that provides a basis for a discussion to define a tool that can be easily used by the bio-logging community. However, the goal to eventually enable biologists to generate animal trackers without the support of engineers is ambitious. A future study should investigate how this can be facilitated by a user interface tailored to the needs of biologists and able to handle complex projects. Since the current design automation prototype runs locally on a computer, the development of a web interface that can access the code online is a further possibility for future work. The code of the design automation prototype could be made available online via a CAD server and the *RhinoCompute API*, the *Shapediver* (ShapeDiver GmbH, Austria) platform or implemented using *Paramate* (trinckle 3D GmbH, Germany). In addition, it needs to be clarified how databases and algorithms can be implemented and maintained. The current version of the code is open-sourced in a public GitHub repository and a web tool could be hosted by initiatives such as Movebank.org, companies, or networks of research institutions. It is particularly important that the tool is maintained and further developed to have a long-term impact that realizes the vision described. Whether such a platform can be economically viable and useful depends largely on the response from the bio-logging community and the demand for such mass-customized species-specific animal trackers.

## Conclusions

This paper presents a vision of how species-specific animal trackers can be customized faster in the future, while also improving device quality and interdisciplinary collaboration. The vision includes automation of engineering processes with the two main functions of (i) selecting electronic components such as circuit board, battery, and solar cell and (ii) generating the 3D geometries of the housings in a CAD program. The vision also includes the development of a web-based user interface for biologists that allows them to automatically design an experiment-tailored animal tracker based on biologically relevant parameters and then start the automated engineering processes. Another user interface allows the manual adjustment of engineering design parameters by either biologists or engineers. Such a tool can significantly reduce development time, reduce the number of development iterations, increase the reusability of validated design features, and reduce the dependency of biologists on engineers. To demonstrate this, this paper presents a design automation prototype for the automated generation of bio-logger housings. The prototype follows a design automation approach and demonstrates the core features of the vision. Users can use number sliders to make manual adjustments with many different design parameters such as wall thickness, radii, or other geometric sizes, if necessary. Within the current pilot study, animal tracker housings for three different species were successfully autogenerated and physical prototypes were additive manufactured (3D printed). The generation of geometries takes less than 50 seconds for all use cases, which emphasizes the reduction in development time.

Looking forward, the platform can be expanded into a full-scale, user-ready system through close collaboration between biologists and engineers in academia and industry to refine practical requirements and validate new features. To support this development, future work may incorporate a curated component database, an automated component selector, and extended housing-generation capabilities for additional attachment methods and species. Deploying the tool as a web-based service with online computation and a reliable manufacturing workflow would further enhance accessibility and reproducibility. Iterative usability studies and field tests will be essential to ensure that the system meets real-world needs and can ultimately be operated by biologists with minimal engineering support. If the potential of the presented platform can be fully exploited, it can become an important building block in the realization of a truly scalable internet of animals.

## Supporting information

S1 Fig Computation time for the three use cases categorized by the five design steps. The software is executed on a business notebook equipped with an Intel Core i7-8565U CPU (4 cores, 8 threads, 1.80 GHz base clock, 4.6 GHz turbo boost), 16 GB DDR4 RAM (2133 MHz) and no dedicated GPU. The system runs on Windows 11 and the algorithms are based on the RhinoCommon version 8.9.24194.18121.
(TIF)

S2 File Demonstration video of the design automation prototype. This video provides an insight into the work with the design automation prototype by guiding through all steps from component selection to export geometries.
(MOV)

## References

[1] Kays R, Crofoot MC, Jetz W, Wikelski M. ECOLOGY. Terrestrial animal tracking as an eye on life and planet. Science. 2015;348(6240):aaa2478. doi: 10.1126/science.aaa2478 26068858

[2] Nathan R, Getz WM, Revilla E, Holyoak M, Kadmon R, Saltz D, et al. A movement ecology paradigm for unifying organismal movement research. Proc Natl Acad Sci U S A. 2008;105(49):19052–9. doi: 10.1073/pnas.0800375105 19060196 PMC2614714

[3] Cooke SJ, Hinch SG, Wikelski M, Andrews RD, Kuchel LJ, Wolcott TG, et al. Biotelemetry: a mechanistic approach to ecology. Trends Ecol Evol. 2004;19(6):334–43. doi: 10.1016/j.tree.2004.04.003 16701280

[4] Gould LA, Manning AD, McGinness HM, Hansen BD. A review of electronic devices for tracking small and medium migratory shorebirds. Anim Biotelemetry. 2024;12(1). doi: 10.1186/s40317-024-00368-z

[5] Lahoz-Monfort JJ, Magrath MJL. A Comprehensive Overview of Technologies for Species and Habitat Monitoring and Conservation. Bioscience. 2021;71(10):1038–62. doi: 10.1093/biosci/biab073 34616236 PMC8490933

[6] Buchan C, Gilroy JJ, Catry I, Hewson CM, Atkinson PW, Franco AMA. Combining remote sensing and tracking data to quantify species’ cumulative exposure to anthropogenic change. Glob Chang Biol. 2023;29(23):6679–92. doi: 10.1111/gcb.16974 37812027 PMC10946810

[7] Hebblewhite M, Haydon DT. Distinguishing technology from biology: a critical review of the use of GPS telemetry data in ecology. Philos Trans R Soc Lond B Biol Sci. 2010;365(1550):2303–12. doi: 10.1098/rstb.2010.0087 20566506 PMC2894965

[8] Kays R, Wikelski M. The Internet of Animals: what it is, what it could be. Trends Ecol Evol. 2023;38(9):859–69. doi: 10.1016/j.tree.2023.04.007 37263824

[9] Toledo S, Mendel S, Levi A, Vortman Y, Ullmann W, Scherer L-R, et al. Vildehaye: A Family of Versatile, Widely-Applicable, and Field-Proven Lightweight Wildlife Tracking and Sensing Tags. 2022 21st ACM/IEEE International Conference on Information Processing in Sensor Networks (IPSN) 2022. doi: 10.1109/IPSN54338.2022.00008

[10] Wild TA, Koblitz JC, Dechmann DKN, Dietz C, Meboldt M, Wikelski M. Micro-sized open-source and low-cost GPS loggers below 1 g minimise the impact on animals while collecting thousands of fixes. PLoS One. 2022;17(6):e0267730. doi: 10.1371/journal.pone.0267730 35767535 PMC9242438

[11] Kölzsch A, Neefjes M, Barkway J, Müskens GJDM, van Langevelde F, de Boer WF, et al. Neckband or backpack? Differences in tag design and their effects on GPS/accelerometer tracking results in large waterbirds. Anim Biotelemetry. 2016;4(1). doi: 10.1186/s40317-016-0104-9

[12] Kunnasranta M, Miettinen E, Melin M, Meller A, Väänänen V-M, Huitu O, et al. The performance of alternative GPS tracking devices: a case report on wild boars (Sus scrofa). Anim Biotelemetry. 2024;12(1). doi: 10.1186/s40317-024-00382-1

[13] Wilson RP, McMahon CR. Measuring devices on wild animals: what constitutes acceptable practice?. Frontiers in Ecology and the Environment. 2006;4(3):147–54. doi: 10.1890/1540-9295(2006)0040147:mdowaw.2.0.co;2

[14] Bowlin MS, Henningsson P, Muijres FT, Vleugels RHE, Liechti F, Hedenström A. The effects of geolocator drag and weight on the flight ranges of small migrants. Methods in Ecology and Evolution. 2010;1(4):398–402. doi: 10.1111/j.2041-210x.2010.00043.x

[15] Wikner J, Rudberg M. Integrating production and engineering perspectives on the customer order decoupling point. International Journal of Operations & Production Management. 2005;25(7):623–41. doi: 10.1108/01443570510605072

[16] Rudberg M, Wikner J. Mass customization in terms of the customer order decoupling point. Production Planning & Control. 2004;15(4):445–58. doi: 10.1080/0953728042000238764

[17] Olhager J. Strategic positioning of the order penetration point. International Journal of Production Economics. 2003;85(3):319–29. doi: 10.1016/s0925-5273(03)00119-1

[18] Mosig T, Karl Grafmüller L, Lehmann C. Business Model Patterns of B2B Mass Customizers: The Case of German Textile SMEs. Int J Ind Eng Manag. 2017;8(3):99–110. doi: 10.24867/ijiem-2017-3-111

[19] Thomassen MK, Alfnes E. Mass Customization Challenges of Engineer-to-Order Manufacturing. Springer Proceedings in Business and Economics. Springer International Publishing. 2016. p. 27–39. doi: 10.1007/978-3-319-29058-4_3

[20] Piller FT. Mass Customization: Reflections on the State of the Concept. Int J Flex Manuf Syst. 2004;16(4):313–34. doi: 10.1007/s10696-005-5170-x

[21] Zipkin P. The limits of mass customization. MIT Sloan Management Review. 2001;42:81–7.

[22] Aldridge HDJN, Brigham RM. Load Carrying and Maneuverability in an Insectivorous Bat: a Test of the 5% “Rule” of Radio-Telemetry. Journal of Mammalogy. 1988;69(2):379–82. doi: 10.2307/1381393

[23] Gosling J, Naim MM. Engineer-to-order supply chain management: A literature review and research agenda. International Journal of Production Economics. 2009;122(2):741–54. doi: 10.1016/j.ijpe.2009.07.002

[24] Fortes CS, Tenera AB, Cunha PF. Engineer-to-Order Challenges and Issues: A Systematic Literature Review of the manufacturing industry. Procedia Computer Science. 2023;219:1727–34. doi: 10.1016/j.procs.2023.01.467

[25] Iakymenko N, Romsdal A, Semini M, Strandhagen JO. Managing engineering changes in the engineer-to-order environment: challenges and research needs. IFAC-PapersOnLine. 2018;51(11):144–51. doi: 10.1016/j.ifacol.2018.08.24930480263

[26] Jünge G, Alfnes E, Nujen B, Emblemsvag J, Kjersem K. Understanding and eliminating waste in Engineer-To-Order (ETO) projects: a multiple case study. Production Planning & Control. 2021;34(3):225–41. doi: 10.1080/09537287.2021.1903279

[27] Williams HJ, Taylor LA, Benhamou S, Bijleveld AI, Clay TA, de Grissac S, et al. Optimizing the use of biologgers for movement ecology research. J Anim Ecol. 2020;89(1):186–206. doi: 10.1111/1365-2656.13094 31424571 PMC7041970

[28] Peterson C, Paasch RK, Ge P, Dietterich TG. Product Innovation for Interdisciplinary Design under Changing Requirements. International Conference on Engineering Design (ICED) 2007:861.

[29] Edmondson AC, Nembhard IM. Product Development and Learning in Project Teams: The Challenges Are the Benefits*. J of Product Innov Manag. 2009;26(2):123–38. doi: 10.1111/j.1540-5885.2009.00341.x

[30] Cannas VG, Gosling J. A decade of engineering-to-order (2010–2020): Progress and emerging themes. International Journal of Production Economics. 2021;241:108274. doi: 10.1016/j.ijpe.2021.108274

[31] Viollet S, Huloux N, Diperi J, Ingargiola J-M, Kato A, Ropert-Coudert Y. Open source bio-logger for monitoring and recording inertial movement. IEEE International Symposium on Intertial Sensors and Systems 2022. doi: 10.1109/INERTIAL53425.2022.9787736

[32] Kunz M, Lone K. Using GPS tracking to determine flight patterns of red-footed boobies (Sula sula) near Palmyra Atoll. 2007.

[33] Sandfort R. Waterproofing DIY VHF transmitter. WildlabsNet 2024. (accessed October 28, 2024). https://www.wildlabs.net/discussion/waterproofing-diy-vhf-transmitter

[34] Weaver SJ, Westphal MF, Taylor EN. Technology wish lists and the significance of temperature-sensing wildlife telemetry. Anim Biotelemetry. 2021;9(1). doi: 10.1186/s40317-021-00252-0

[35] Lange MW, Imsdahl A. Modular Function Deployment: Using Module Drivers to Impart Strategies to a Product Architecture. Advances in Product Family and Product Platform Design. Springer New York. 2013. p. 91–118. doi: 10.1007/978-1-4614-7937-6_4

[36] Lehnerd AP, Meyer MH. The power of product platforms. Simon & Schuster. 1997.

[37] Wild TA. Smart bio-logging technology for the tracking of animal collectives. PhD Thesis. ETH Zürich, 2023. doi: 10.3929/ethz-b-000647599

[38] Bright RJG, Newman C, Finerty GE, Wijers M, Foley CJ, Buesching CD. The effects of energetic expenditure tactics and life-history variability on European badger (Meles meles) ecology. University of Oxford. 2021.

[39] Siguín M, Blanco T, Rossano F, Casas R. Modular E-Collar for Animal Telemetry: An Animal-Centered Design Proposal. Sensors (Basel). 2021;22(1):300. doi: 10.3390/s22010300 35009840 PMC8749898

[40] Gregersen T, Wild TA, Havmøller LW, Møller PR, Lenau TA, Wikelski M, et al. A novel kinetic energy harvesting system for lifetime deployments of wildlife trackers. PLoS One. 2023;18(5):e0285930. doi: 10.1371/journal.pone.0285930 37196042 PMC10191315

[41] Klahn C, Leutenecker B, Meboldt M. Design Strategies for the Process of Additive Manufacturing. Procedia CIRP. 2015;36:230–5. doi: 10.1016/j.procir.2015.01.082

[42] Thompson MK, Moroni G, Vaneker T, Fadel G, Campbell RI, Gibson I, et al. Design for Additive Manufacturing: Trends, opportunities, considerations, and constraints. CIRP Annals. 2016;65(2):737–60. doi: 10.1016/j.cirp.2016.05.004

[43] Gibson I, Rosen D, Stucker B, Khorasani M. Design for Additive Manufacturing. Additive Manufacturing Technologies. Springer International Publishing. 2020. p. 555–607. doi: 10.1007/978-3-030-56127-7_19

[44] Verhagen WJC, Bermell-Garcia P, van Dijk REC, Curran R. A critical review of Knowledge-Based Engineering: An identification of research challenges. Advanced Engineering Informatics. 2012;26(1):5–15. doi: 10.1016/j.aei.2011.06.004

[45] Amadori K, Tarkian M, Ölvander J, Krus P. Flexible and robust CAD models for design automation. Advanced Engineering Informatics. 2012;26(2):180–95. doi: 10.1016/j.aei.2012.01.004

[46] Mulder B, La Rocca G, Schut EJ, Verhagen WJC. A Methodological Approach for the Optimisation of the Product Development Process by the Application of Design Automation. Challenges in European Aerospace 2015.

[47] Kügler P, Dworschak F, Schleich B, Wartzack S. The evolution of knowledge-based engineering from a design research perspective: Literature review 2012–2021. Advanced Engineering Informatics. 2023;55:101892. doi: 10.1016/j.aei.2023.101892

[48] Rigger E, Münzer C, Shea K. Estimating the potential of state of the art design automation - Tasks, methods, and benefits. In: Proceedings of International Design Conference, DESIGN 2016, 2016. 421–32.

[49] Chapman CB, Pinfold M. Design engineering—a need to rethink the solution using knowledge based engineering. Knowledge-Based Systems. 1999;12(5–6):257–67. doi: 10.1016/s0950-7051(99)00013-1

[50] Rocca GL. Knowledge based engineering: Between AI and CAD. Review of a language based technology to support engineering design. Advanced Engineering Informatics. 2012;26(2):159–79. doi: 10.1016/j.aei.2012.02.002

[51] Vidner O, Wehlin C, Wiberg A. Design Automation Systems for the Product Development Process: Reflections from Five Industrial Case Studies. Proc Des Soc. 2022;2:2533–42. doi: 10.1017/pds.2022.256

[52] Oettmeier K, Hofmann E. Impact of additive manufacturing technology adoption on supply chain management processes and components. JMTM. 2016;27(7):944–68. doi: 10.1108/jmtm-12-2015-0113

[53] Biedermann M, Beutler P, Meboldt M. Routing multiple flow channels for additive manufactured parts using iterative cable simulation. Additive Manufacturing. 2022;56:102891. doi: 10.1016/j.addma.2022.102891

[54] Biedermann M, Beutler P, Meboldt M. Automated design of additive manufactured flow components with consideration of overhang constraint. Additive Manufacturing. 2021;46:102119. doi: 10.1016/j.addma.2021.102119

[55] Wiberg A. Towards Design Automation for Additive Manufacturing - A Multidisciplinary Optimization approach. PhD Thesis. Linköping University, 2019. doi: 10.3384/lic.diva-160888

[56] Beutler P, Berger M, Ferchow J, Meboldt M. Enhanced Design Automation for Hydraulic Manifolds Produced Using Additive Manufacturing. Procedia CIRP. 2024;128:162–7. doi: 10.1016/j.procir.2024.06.017

[57] Omidvarkarjan D, Cipriano D, Rosenbauer R, Biedermann M, Meboldt M. Implementation of a design support tool for additive manufacturing using a feature database: an industrial case study. Prog Addit Manuf. 2020;5(1):67–73. doi: 10.1007/s40964-020-00119-5

[58] Tedaldi G, Miragliotta G. The role of Engineering-to-Order machinery manufacturers in future Cloud Manufacturing supply chains: a business case and a strategic perspective. Production Planning & Control. 2021;33(9–10):1011–23. doi: 10.1080/09537287.2020.1837942

[59] Charro A, Schaefer D. Cloud Manufacturing as a new type of Product-Service System. International Journal of Computer Integrated Manufacturing. 2018;31(10):1018–33. doi: 10.1080/0951192x.2018.1493228

[60] Wild TA, Wikelski M, Tyndel S, Alarcón‐Nieto G, Klump BC, Aplin LM, et al. Internet on animals: Wi‐Fi‐enabled devices provide a solution for big data transmission in biologging. Methods Ecol Evol. 2022;14(1):87–102. doi: 10.1111/2041-210x.13798

[61] Wild TA, Wilbs G, Dechmann DKN, Kohles JE, Linek N, Mattingly S, et al. Time synchronisation for millisecond-precision on bio-loggers. Mov Ecol. 2024;12(1):71. doi: 10.1186/s40462-024-00512-7 39468685 PMC11520525

[62] Strandburg-Peshkin A, Farine DR, Couzin ID, Crofoot MC. GROUP DECISIONS. Shared decision-making drives collective movement in wild baboons. Science. 2015;348(6241):1358–61. doi: 10.1126/science.aaa5099 26089514 PMC4801504

[63] Lambertucci SA, Navarro J, Sanchez Zapata JA, Hobson KA, Alarcón PAE, Wiemeyer G, et al. Tracking data and retrospective analyses of diet reveal the consequences of loss of marine subsidies for an obligate scavenger, the Andean condor. Proc Biol Sci. 2018;285(1879):20180550. doi: 10.1098/rspb.2018.0550 29848650 PMC5998103

[64] Anderson D, Arkumarev V, Bildstein K, Botha A, Bowden C, Davies M, et al. A practical guide to methods for attaching research devices to vultures and condors. Vulture News. 2020;78a. doi: 10.17863/CAM.58032

[65] Cai C, Tey WS, Chen J, Zhu W, Liu X, Liu T, et al. Comparative study on 3D printing of polyamide 12 by selective laser sintering and multi jet fusion. Journal of Materials Processing Technology. 2021;288:116882. doi: 10.1016/j.jmatprotec.2020.116882

[66] Tan LJ, Zhu W, Zhou K. Recent Progress on Polymer Materials for Additive Manufacturing. Adv Funct Materials. 2020;30(43). doi: 10.1002/adfm.202003062

[67] Adach M, Sokołowski P, Piwowarczyk T, Nowak K. Study on Geometry, Dimensional Accuracy and Structure of Parts Produced by Multi Jet Fusion. Materials (Basel). 2021;14(16):4510. doi: 10.3390/ma14164510 34443032 PMC8398662

[68] Singh AP, Pervaiz S. Current Status and Prospects of Multi-Jet Fusion (MJF) based 3D Printing Technology. Proceedings of the ASME 2021 International Mechanical Engineering Congress and Exposition 2021. doi: 10.1115/IMECE2021-73547

