# Supplementary figures and images for: Toward mass customization of animal trackers by design automation
Source: PLoS One. 2026 Feb 4;21(2):e0342071. doi: 10.1371/journal.pone.0342071 (PMC12871966; doi:10.1371/journal.pone.0342071)

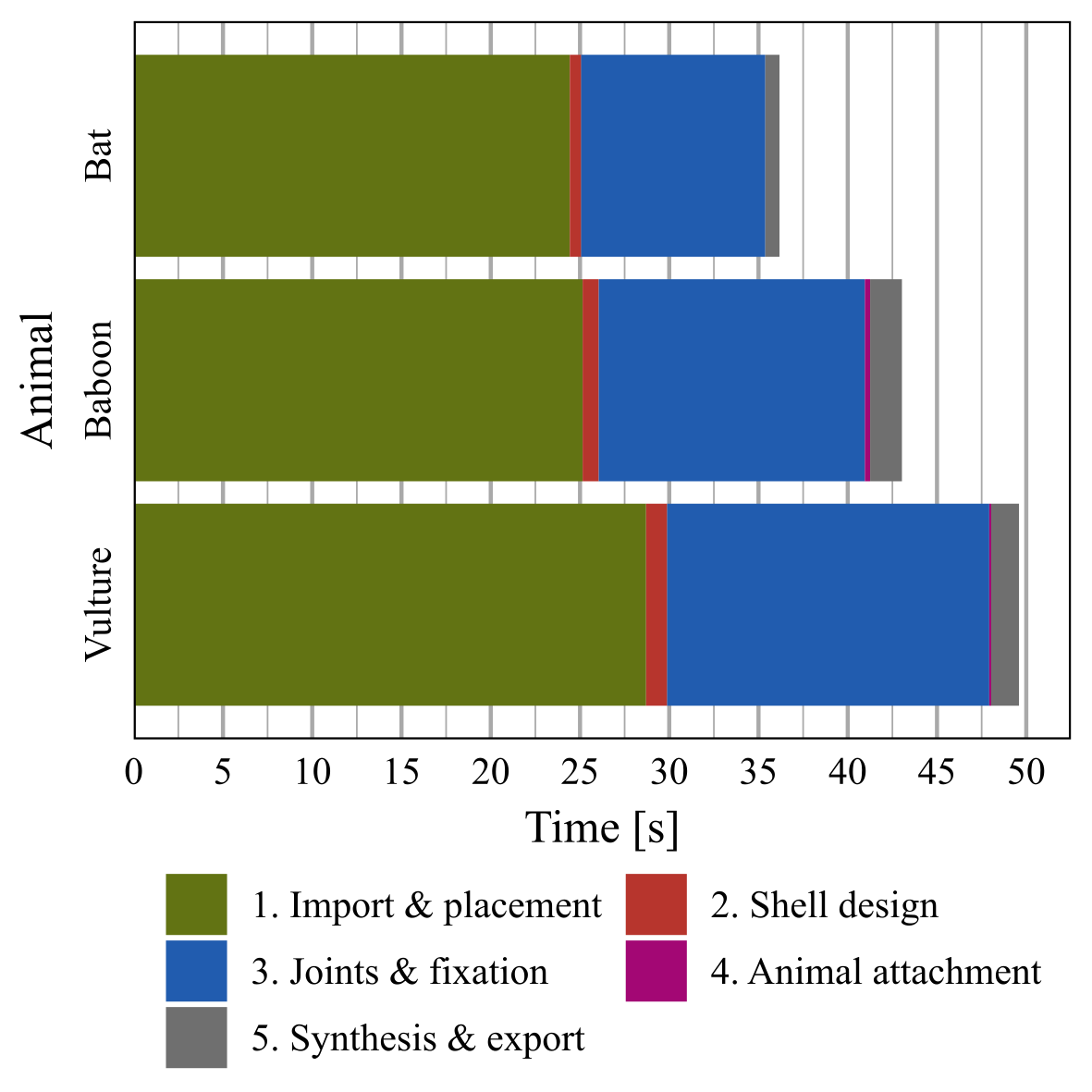

Supplement: S1 Fig — The software is executed on a business notebook equipped with an Intel Core i7-8565U CPU (4 cores, 8 threads, 1.80 GHz base clock, 4.6 GHz turbo boost), 16 GB DDR4 RAM (2133 MHz) and no dedicated GPU. The system runs on Windows 11 and the algorithms are based on the RhinoCommon version 8.9.24194.18121. (TIF) [file pone.0342071.s001.tif]
